# Supplementary material for: MetaRibo-Seq measures translation in microbiomes
Source: Nat Commun. 2020 Jun 29;11:3268. doi: 10.1038/s41467-020-17081-z (PMC7324362; doi:10.1038/s41467-020-17081-z)
Supplement: Supplementary file 10 — Supplementary Data 7 [file 41467_2020_17081_MOESM10_ESM.zip › File2/Confidence_High_Taxonomy/35225_out.krona.html]

Javascript must be enabled to view this page.

members
magnitude
magnitudeUnassigned
count
unassigned
taxon
rank

35225\_out

13

2
superkingdom
13

13
phylum
976

class
200643
13

13
order
171549

family
171552
13

genus
838
13

11

SRS015782\_contig\_number\_39932SRS016541\_contig\_number\_16785SRS017307\_contig\_number\_30891SRS018888\_contig\_number\_811SRS023914\_contig\_number\_12428SRS047044\_contig\_number\_16148SRS049896\_contig\_number\_14679SRS064757\_contig\_number\_20526SRS104036\_contig\_number\_2355SRS1041116\_contig\_number\_3263SRS144506\_contig\_number\_55106
species
165179

59823
species

SRS100907\_contig\_number\_contig-100\_2717.65159
1


SRS054059\_contig\_number\_3987
1
1263102
species
